# Association Between Early *Helicobacter pylori* Eradication and a Lower Risk of Recurrent Complicated Peptic Ulcers in End-Stage Renal Disease Patients

**DOI:** 10.1097/MD.0000000000000370

**Published:** 2015-01-09

**Authors:** Shen-Shong Chang, Hsiao-Yun Hu

**Affiliations:** From the Division of Gastroenterology (S-SC), Department of Internal Medicine, Taipei City Hospital Yang-Ming Branch; School of Medicine (S-SC); Institute of Public Health & Department of Public Health (H-YH), National Yang-Ming University; and Department of Education and Research (H-YH), Taipei City Hospital, Taipei, Taiwan.

## Abstract

End-stage renal disease (ESRD) patients exhibit an increased incidence of peptic ulcer disease. *Helicobacter pylori* plays a central role in the development of peptic ulcers. The effect of early *H pylori* eradication on the recurrence of complicated peptic ulcer disease in ESRD patients remains unclear. The aim of the present study was to explore whether early *H pylori* eradication therapy in ESRD patients can reduce the risk of recurrent complicated peptic ulcers.

We conducted a population-based cohort study and recruited patients with ESRD who had developed peptic ulcers. We categorized patients into early (time lag ≦120 days after peptic ulcer diagnosis) and late *H pylori* eradication therapy groups. The Cox proportional hazards model was used. The endpoint was based on hospitalization for complicated recurrent peptic ulcers.

The early and late *H pylori* eradication therapy groups consisted of 2406 and 1356 ESRD patients, respectively, in a time lag of 120 days. After adjusting for possible confounders, the early eradication group exhibited a lower rate of complicated recurrent peptic ulcer disease (hazard ratio [HR] = 0.76, 95% confidence interval [CI] = 0.64–0.91, *P* = 0.003) in a time lag of ≦120 days, but a similar rate of complicated recurrent peptic ulcer disease in time lags of ≦1 year (HR = 0.97, 95% CI 0.79–1.19, *P* = 0.758) and 2 years (HR = 1.11, 95% CI 0.86–1.44, *P* = 0.433) compared with the late eradication group.

We recommend administering *H pylori* eradication within 120 days after peptic ulcer diagnosis to *H pylori* infected ESRD patients who have developed peptic ulcers.

## INTRODUCTION

End-stage renal disease (ESRD) patients exhibit a higher incidence of peptic ulcer disease than do patients without renal disease.^1^*Helicobacter pylori* plays a central role in the development of peptic ulcers.^2^ Therefore, physicians must consider these 2 factors when treating patients with upper gastrointestinal disease. The effect of early *H pylori* eradication on the recurrence of complicated peptic ulcer disease, which is the primary cause of threat to life and mortality, remains unclear.

Chen et al^3^ reported the prevalence of *H pylori* infection (90%) to be increased among people in the general population who have developed peptic ulcer disease. Hopkins et al^4^ reported that the recurrence of peptic ulcers can markedly decrease from 70% to 10% or lower following *H pylori* eradication. However, Kang et al^5^ reported a 29% *H pylori* infection rate among ESRD patients who developed peptic ulcers. These results imply the diverse gastric environment of ESRD patients. Factors such as reductions in mucosa prostaglandin,^1^ hypergastrinemia,^6^ drugs such as nonsteroidal anti-inflammatory drugs (NSAIDs),^7^ and systemic/local circulatory failure^8^ influence the onset of recurrent peptic ulcer disease in ESRD patients. Clarifying the role of *H pylori* eradication in the pathogenesis of recurrent peptic ulcer bleeding (PUB) and perforations in ESRD patients is crucial.

We divided patients into an early eradication therapy group and a late eradication therapy group. Our study explored whether early *H pylori* eradication therapy in ESRD patients reduces the risk of recurrent complicated peptic ulcer disease.

## MATERIALS AND METHODS

### Ethics Statement

The National Health Insurance Database (NHIRD) is a secondary database. The information on the identity of subjects from the database was scrambled before it was released for research purpose. The privacy and confidentiality of all beneficiaries were safeguarded by the Taiwan National Health Research Institute (NHRI). The data are publicly available. Patient records/information was anonymized and de-identified prior to analysis. In this study, ethics approval was approved by the NHRI and the Institutional Review Board (IRB) of Taipei City Hospital (IRB No.: TCHIRB-1020424-E). Written consent was waived by the approving IRB.

### Study Population

This nationwide cohort study was based on patient data obtained from the NHIRD, which is managed by the Taiwan NHRI. The NHIRD contains health care data on 99% of the population of Taiwan (approximately 23 million people).^9^

### Study Patients

**FIGURE 1:**
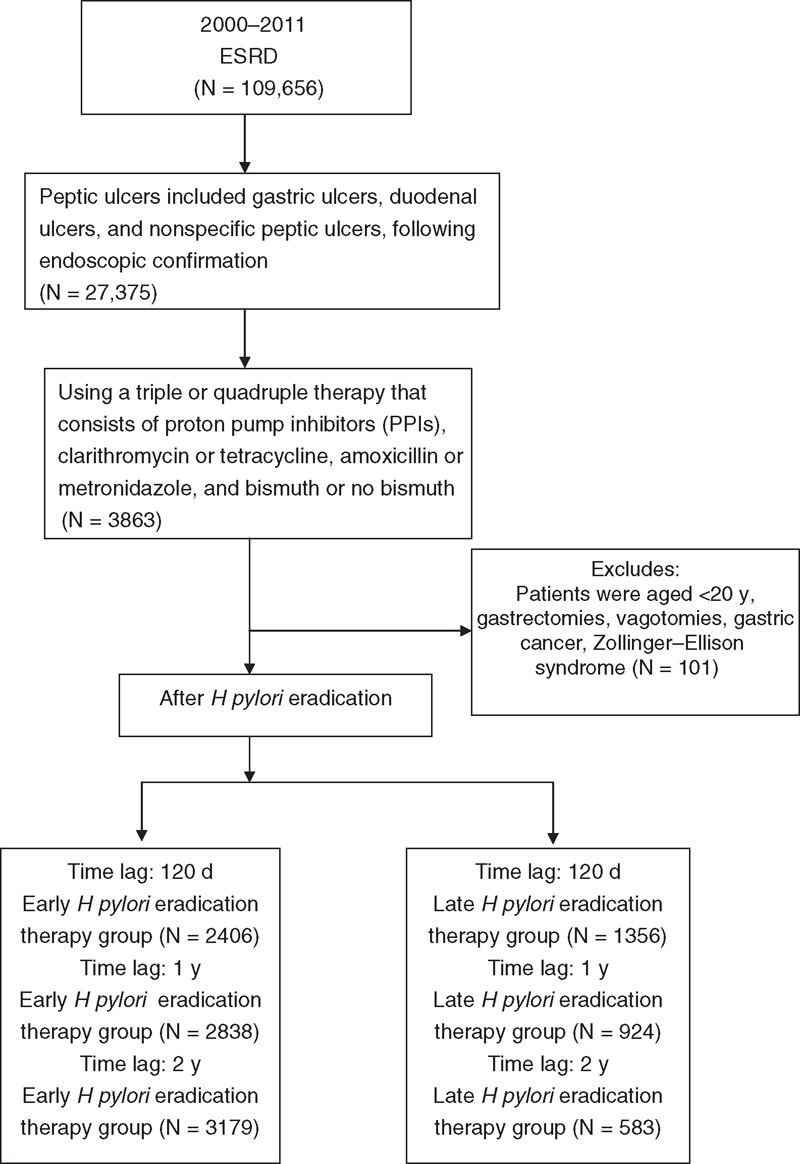
Flowchart depicting the selection of the participants. ESRD = end-stage renal disease.

In this population-based cohort study, patients with ESRD receiving regular hemodialysis who were diagnosed with peptic ulcers between 2000 and 2011 constituted the study cohort. The definition of peptic ulcers included gastric ulcers (International Classification of Diseases, Ninth Revision, Clinical Modification [ICD-9-CM]: 531), duodenal ulcers (ICD-9-CM: 532), and nonspecific peptic ulcers (ICD-9-CM: 533), and diagnoses were confirmed through endoscopic examination. Figure 1 shows a flowchart of the patient selection process.

### Definition of End-Stage Renal Disease

In Taiwan, ESRD patients requiring dialysis can apply to receive a catastrophic illness card. Cardholders are exempt from the cost sharing required by the National Health Insurance (NHI) program. ESRD patients were defined as patients having a catastrophic illness card, a minimum of 10 outpatient claims, and a diagnosis defined according to ICD-9-CM diagnosis code 585.

### Definitions of the Early and Late *H pylori* Eradication Groups

Measuring from the time of peptic ulcer diagnosis to the time of *H pylori* eradication therapy, we classified patients as being either in the early *H pylori* eradication therapy group (time lag ≦120 days after peptic ulcer diagnosis) or in the late *H pylori* eradication therapy group.^10^ The *H pylori* status was determined using rapid urease tests or a histological assessment using hematoxylin and eosin staining. The *H pylori* eradication therapy was a triple or quadruple therapy consisting of proton pump inhibitors (PPIs), clarithromycin or tetracycline, amoxicillin or metronidazole, and bismuth or no bismuth.

### Definition of Peptic Ulcer History

All ESRD patients with peptic ulcers endoscopically diagnosed between 1997 and 1999 according to ambulatory care and inpatient discharge records were defined as having a peptic ulcer history.

### Definition of Comorbidities

Comorbidities were recorded for patients who were identified in either inpatient discharge records or 3 or more ambulatory care claims according to ICD-9-CM codes recorded between January 1, 1996 and the index date. The comorbidities identified in our cohort and the corresponding ICD-9-CM diagnosis codes are listed as follows: diabetes mellitus, ICD-9-CM: 250; coronary heart disease, ICD-9-CM: 405, 410–414, and 428; cerebral vascular disease (CVD), ICD-9-CM: 430–438; dyslipidemia, ICD-9-CM: 272.xx; and liver cirrhosis, ICD-9-CM: 571.2, 571.5, and 571.6.

### Medication Users

We defined patients who received at least 1 prescription of PPIs, histamine receptor-2 blockers (H_2_-blockers), aspirin, NSAIDs, cyclooxygenase-2 (COX-2)-specific inhibitors, steroids, clopidogrel, ticlopidine, and warfarin within 28 days prior to the end of the observation period as medication users.

### Endpoint

Based on inpatient discharge records, hospitalization for complicated recurrent peptic ulcer disease with hemorrhages or perforations following endoscopic confirmation after *H pylori* eradication between 2000 and 2010 was defined using the following ICD-9-CM codes: 531.0, 531.1, 531.2, 531.4, 531.5, 531.6, 532.0, 532.1, 532.2, 532.4, 532.5, 532.6, 533.0, 533.1, 533.2, 533.4, 533.5, and 533.6.

### Statistical Analysis

Categorical variables are presented as percentages. The χ^2^ test was used for categorical data. Continuous variables were expressed as means ± standard deviation (SD). The Cox proportional hazards model was used. Risk estimations are presented as hazard ratios (HRs) determined using a 95% confidence interval (CI). *P* < 0.05 indicated a statistically significant difference. All statistical analyses were performed using an SAS statistical package (SAS System for Windows, Version 9.2; SAS Institute, Cary, NC).

## RESULTS

### Demographic Data

The early and late *H pylori* eradication therapy groups consisted of 2406 and 1356 ESRD patients in a time lag of 120 days. The demographic data are presented in Table 1. A significantly lower percentage of patients in the early *H pylori* eradication therapy group with a peptic ulcer history (*P* = 0.042) used PPIs (*P* < 0.001), H_2_-blockers (*P* < 0.001), COX-2-specific inhibitors (*P* = 0.013), and ticlopidine (*P* = 0.021) than did patients in the late *H pylori* eradication therapy group. In addition, the early *H pylori* eradication therapy group included a significantly higher percentage of male (*P* = 0.009) and CVD (*P* = 0.020) patients compared with the late *H pylori* eradication therapy group. The average follow-up durations were 4.56 ± 2.97 years in the early *H pylori* eradication therapy group and 3.65 ± 2.63 years in the late *H pylori* eradication therapy group (Table 1).

**TABLE 1:**
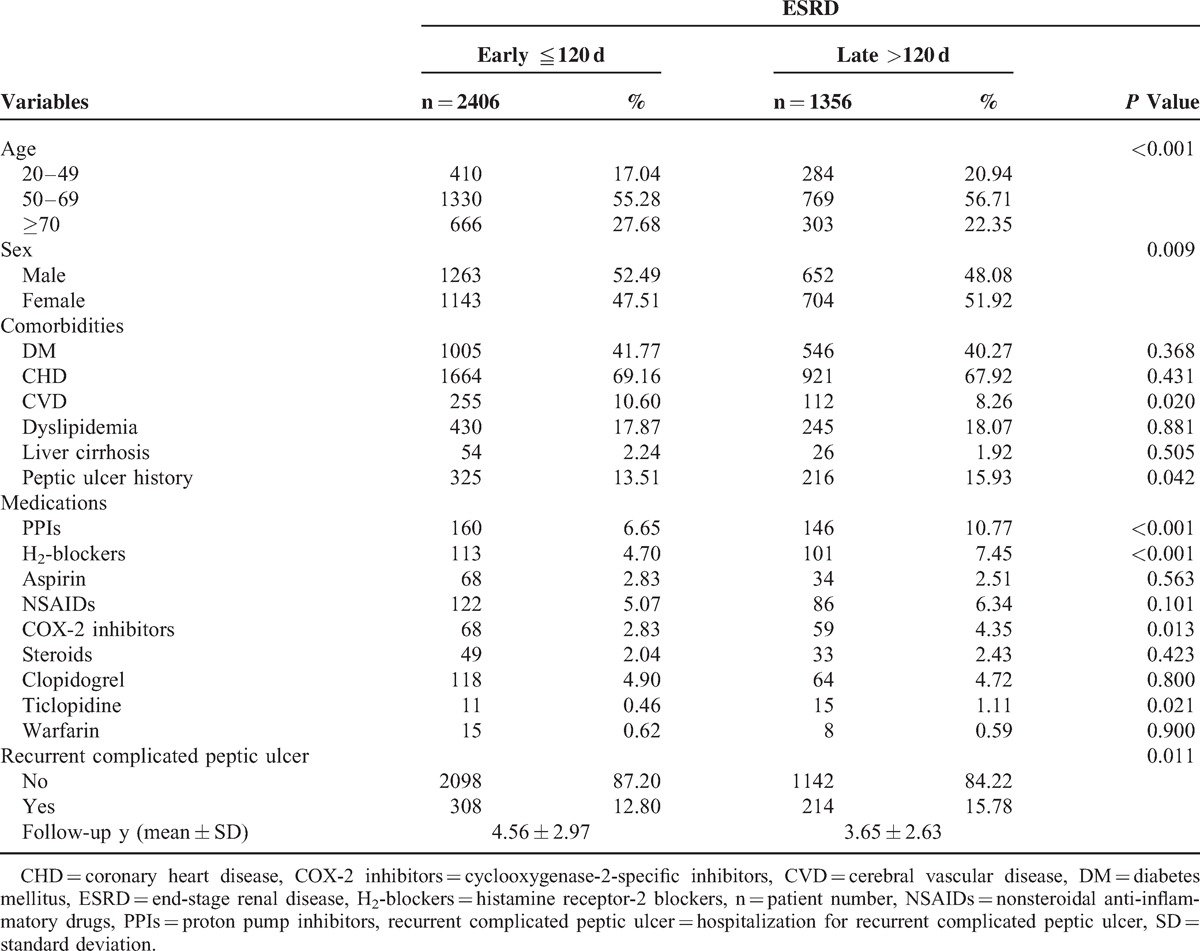
Demographic Characteristics of ESRD Patients

### Multivariate Analysis

After we adjusted for possible confounders, the results of the Cox proportional hazards model analysis indicated that the early *H pylori* eradication therapy group exhibited a lower rate of complicated recurrent peptic ulcer disease (HR = 0.76, 95% CI 0.64–0.91, *P* = 0.003) in a time lag of ≦120 days (Table 2), but a similar rate of complicated recurrent peptic ulcer disease (HR = 0.97, 95% CI 0.79–1.19, *P* = 0.758) in time lags of ≦1 year (Table 3) (HR = 1.11, 95% CI 0.86–1.44, *P* = 0.433) and 2 years (Table 3) compared with the late *H pylori* eradication therapy group.

**TABLE 2:**
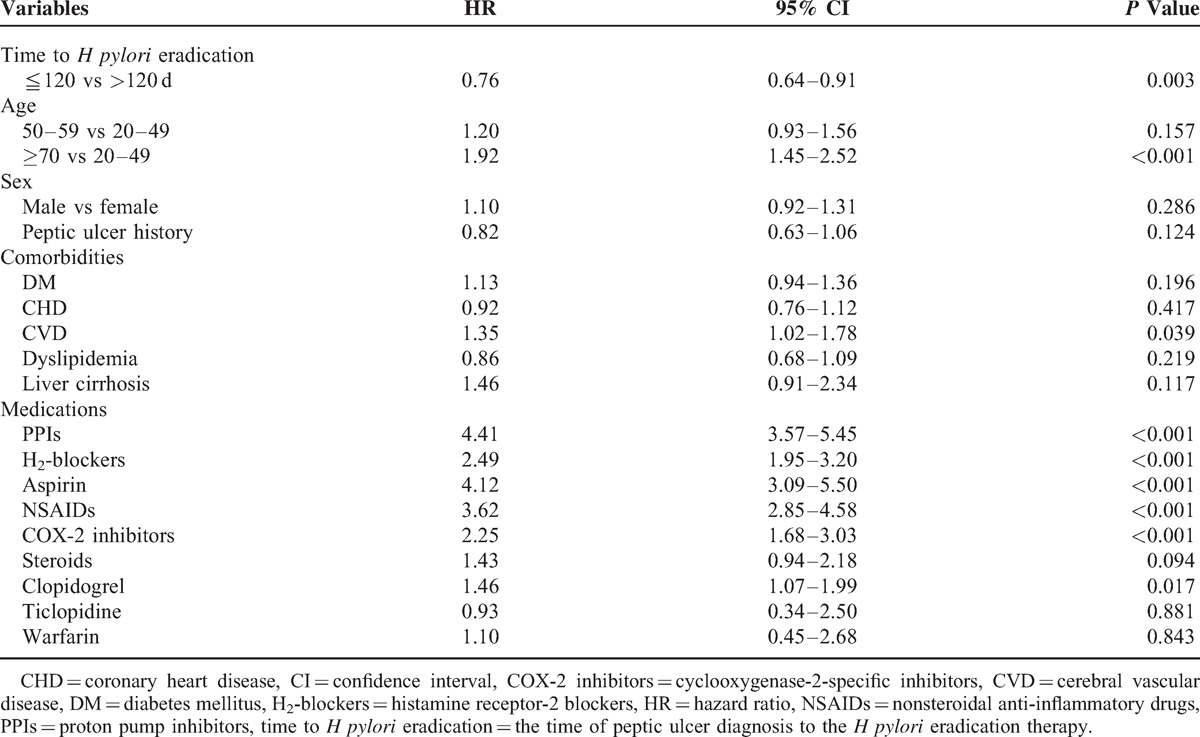
Multivariate Cox Regression Analysis for Prediction of Recurrent Complicated Peptic Ulcers With Time Lag of ≦120 Days in the Overall Study Group

**TABLE 3:**
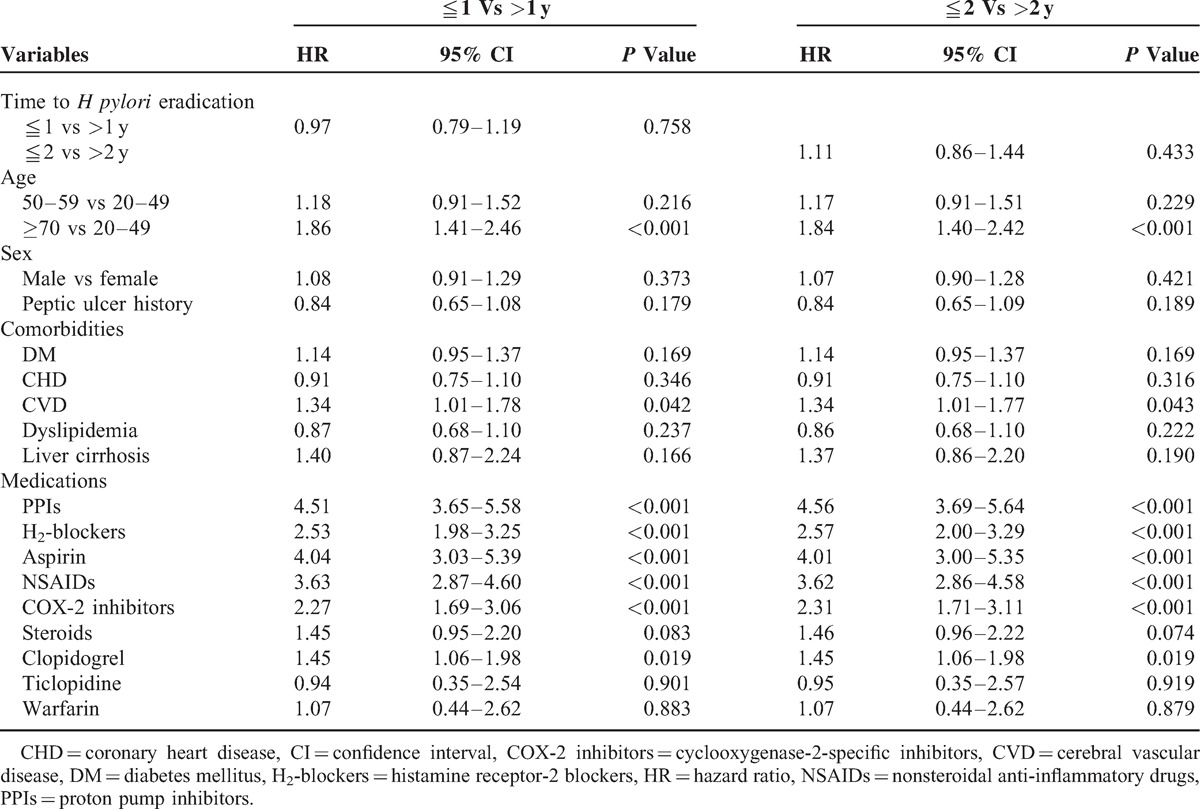
Multivariate Cox Regression Analysis for Prediction of Recurrent Complicated Peptic Ulcers With the Time Lag of ≦1 and 2 Years in the Overall Study Group

### Relative Risk of Complicated Peptic Ulcer Disease

The Cox proportional hazards analysis revealed that patients who were 70 years of age and older (HR = 1.92, 95% CI 1.45–2.52, *P* < 0.001) exhibited a significantly higher risk for complicated recurrent peptic ulcer disease than did patients who were 20 to 49 years of age. In addition, PPIs (HR = 4.41, 95% CI 3.57–5.45, *P* < 0.001), H_2_-blockers (HR = 2.49, 95% CI 1.95-3.20, *P* < 0.001), aspirin (HR = 4.12, 95% CI 3.09–5.50, *P* < 0.001), NSAIDs (HR = 3.62, 95% CI 2.85–4.58, *P* < 0.001), COX-2-specific inhibitors (HR = 2.25, 95% CI 1.68–3.03, *P* < 0.001), and clopidogrel (HR = 1.46, 95% CI 1.07–1.99, *P* = 0.017) were independent risk factors for complicated recurrent peptic ulcer disease.

### Combined Effects of *H pylori* Eradication Therapy and Nonsteroidal Anti-Inflammatory Drug Use on Complicated Peptic Ulcer Disease

**FIGURE 2:**
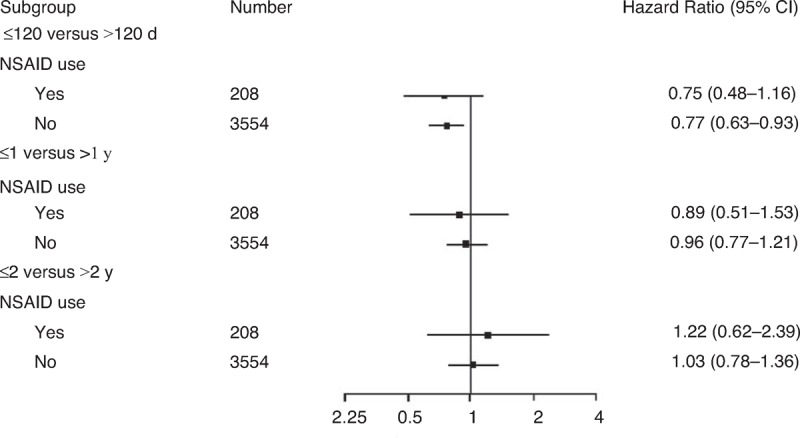
Multivariate stratified Cox proportional hazards model analysis for predicting hospitalization with complicated recurrent peptic ulcers according to NSAID use (it was adjusted for all other factors). NSAID = nonsteroidal anti-inflammatory drug.

An analysis stratified according to NSAID use indicated that patients who did not use NSAIDs in the early *H pylori* eradication therapy group were at a reduced risk of complicated peptic ulcer disease in a time lag of ≦120 days (HR = 0.77, 95% CI 0.63–0.93, *P* = 0.008), compared with the late *H pylori* eradication therapy group. However, patients who did not use NSAIDs in the early *H pylori* eradication therapy group were at a similar risk of complicated recurrent peptic ulcer disease in time lags of ≦1 year (HR = 0.96, 95% CI 0.77–1.21, *P* = 0.734) or 2 years (HR = 1.03, 95% CI 0.78–1.36, *P* = 0.846), compared with the late *H pylori* eradication therapy group (Figure 2).

## DISCUSSION

Our data indicated that early *H pylori* eradication therapy is associated with a reduced risk of recurrent complicated peptic ulcers in ESRD patients in a time lag of 120 days, but no association was observed in a time lag of 1 or 2 years. In addition, patients who did not receive NSAIDs in the early eradication group were at a lower risk of complicated peptic ulcer disease in a time lag of 120 days.

The timing of eradication is a crucial concern. According to the NHI policy in Taiwan, using PPIs and H_2_-blockers is strictly limited to treating endoscopic peptic ulcer patients for 4 months. Therefore, we defined the early *H pylori* eradication therapy group as patients for whom therapy was delayed for fewer than 120 days.^10^ However, the definition of time lag is arbitrary; therefore, we also analyzed the effects of a time lag of ≦1^11^ and 2 years, and obtained different results for these time lags (HR = 0.97, *P* = 0.758; HR = 1.11, *P* = 0.433, respectively).

Sugimoto et al^8,12^ have reported that initiating hemodialysis treatment triggers a decrease in the prevalence of *H pylori* infection. In addition, receiving equal to or <4 years of dialysis treatment had naturally cured *H pylori* infection, thus supporting the practice of administering eradication therapy to *H pylori* infected dialysis patients, particularly those receiving dialysis for 5 years or more. In contrast to Sugimoto et al, our current study revealed that early *H pylori* eradication within 120 days after peptic ulcer diagnosis can reduce recurrent complicated peptic ulcer disease in *H pylori* infected ESRD patients; otherwise it afforded no benefit.

Luo et al^10^ reported that patients with ESRD receiving hemodialysis exhibited a high risk of PUB. A crucial question is whether *H pylori* eradication therapy is necessary for *H pylori* infected dialysis patients. Although *H pylori* eradication is unequivocally effective in preventing peptic ulcer recurrence in the general population,^13^ such effectiveness has not been established in patients with ESRD. Tseng et al^1^ conducted a prospective study in a single hospital and reported that *H pylori* eradication in ESRD patients reduces recurrent peptic ulcer disease. In addition, there is a higher recurrent peptic ulcer rate after successful *H pylori* eradication in *H pylori* infected ESRD patients, compared with that in the general population. However, the methodology applied in this study differs from that applied by Tseng et al, who used a small sample size and a shorter duration of follow-up (2 years) and excluded ulcerogenic medications and focused on recurrent peptic ulcer disease. By contrast, we conducted an 11-year national longitudinal study to analyze the ulcerogenic medication use of ESRD patients who were hospitalized for recurrent complicated peptic ulcer disease, including PUB and perforation.

The *H pylori* status of our patients in the NHIRD was unconfirmed. Patients who received *H pylori* eradication from 1997 to 1999 were excluded from our study, and we did not examine the results of *H pylori* eradication therapy that occurred before 1997. These patients may have received earlier types of *H pylori* eradication therapy or an initial peptic ulcer diagnosis without a concurrent *H pylori* infection. Therefore, we did not analyze ESRD patients who received only antisecretory therapy and no eradication therapy after endoscopic confirmation. Cameron et al^14^ reported a low annual incidence rate of true *H pylori* reinfection of approximately 0.4%. In our study, *H pylori* persisted in the stomach mucosa of the ESRD patients before eradication therapy intervention during the study period.

The use of ulcerogenic agents was determined to be an independent risk factor for recurrent complicated peptic ulcer disease in our current study. The use of ulcerogenic medications such as aspirin (*P* = 0.256) and NSAIDs (*P* = 0.574) in our early and late *H pylori* eradication therapy groups was similar, and the rate of COX-2-specific inhibitor use was lower (*P* = 0.013) in the early eradication group than in the late eradication group (Table 1). Therefore, ulcerogenic medication use was unlikely to bias our results. In our current study, patients who use NSAIDs in the early *H pylori* eradication therapy group were at a similar risk of complicated recurrent peptic ulcer disease in time lags of ≦120 days (HR = 0.75, 95% CI 0.48–1.16, *P* = 0.195), 1 year (HR = 0.89, 95% CI 0.51–1.53, *P* = 0.668), or 2 years (HR = 1.22, 95% CI 0.62–2.39, *P* = 0.572), compared with those in the late *H pylori* eradication therapy group (Figure 2). Therefore, NSAID use is superior to early *H pylori* eradication therapy for the case of recurrent complicated peptic ulcers in *H pylori* infected ESRD patients.

The use of gastroprotective agents, such as prophylactic medications used to treat peptic ulcers, is not supported by the NHI program. However, the costs of H_2_-blockers and PPIs are low; most of these medications cost less than US$0.25 and $0.8 per tablet, respectively. ESRD patients in this study received prophylactic PPIs and H_2_-blockers to reduce the recurrence of peptic ulcers according to physicians’ decision. In addition, we observed an increased risk of recurrent complicated peptic ulcer disease in patients using PPIs (HR = 4.41, *P* < 0.001) and H_2_-blockers (HR = 2.49, *P* < 0.001). However, prophylactic acid suppression medications such as PPIs (6.65% vs 10.77%, *P* < 0.001) and H_2_-blockers (4.70% vs 7.45%, *P* < 0.001) were used in the early and late groups, respectively. Therefore, it would have caused the exact effect of early eradication on recurrent complicated peptic ulcer disease in ESRD patients to be underestimated and did not bias the results.

Our data indicated that only 3863 patients received *H pylori* eradication in 27,375 confirmed gastroscopic peptic ulcer cases. In addition to NSAID use,^15^ idiopathic peptic ulcers, ESRD,^15^ and other comorbidities^16^ related to *H pylori* negative peptic ulcers, numerous ESRD patients who developed peptic ulcer disease delayed *H pylori* diagnostic testing and eradication therapy, which is unlikely to be aggressive *H pylori* testing and eradication in a prospective study^17^ or guideline recommendations.^18^ In addition, we observed a higher rate of recurrent complicated peptic ulcer disease in elderly ESRD patients (Table 2). Effective therapy should be sought to reduce this excessive risk in critically ill and aged patients. Thus, we recommend determining and eradicating *H pylori* as soon as possible, at least within 120 days after peptic ulcer diagnosis to prevent the development of a life-threatening episode.

Our study had several limitations. First, our observations were based on the risk of hospitalization for complicated peptic ulcer disease in ESRD patients. Therefore, caution must be taken in extrapolating our results to other populations, such as those with uncomplicated peptic ulcers, gastric erosions with hemorrhaging, and chronic kidney disease that is not treated using dialysis. Second, the *H pylori* status after eradication therapy is not provided in the NHIRD. However, a recent multicenter study in Taiwan^19^ reported a PPI-based *H pylori* eradication rate of approximately 87.1%. Tseng et al (95%)^1^ and Kang et al (81.8%)^5^ reported a similar PPI-based *H pylori* eradication rate in ESRD patients. Our study examined only peptic ulcer patients using PPI-based *H pylori* eradication therapy; moreover, both cohorts in our study were constructed from the same population over the same time period. In addition, we obtained lower second *H pylori* eradication rates of 7.98% (192/2406) in the early group and 7.30% (99/1356) in the late group during the 11-year period (data not shown). Therefore, the eradication failure rates in our early and late *H pylori* eradication therapy groups should have been similar. Third, establishing a standard definition of endpoint is crucial. Therefore, we analyzed only the risk of hospitalization for complicated recurrent peptic ulcers by using data from inpatient medical databases. The Bureau of the NHI randomly samples the claims data from every hospital and reviews charts to verify diagnostic validity. ICD-9-CM coding was strictly audited for the purpose of reimbursement. Finally, testing for *H pylori* is affected by the concomitant use of medications such as NSAIDs, aspirin, and PPIs. Therefore, ESRD patients who developed peptic ulcers might have been misdiagnosed as having a false-negative *H pylori* infection. Because we focused on early and late *H pylori* eradication therapy, limitations such as false- or true-negative *H pylori* infection diagnoses were unlikely to bias our results.

In conclusion, early *H pylori* eradication is associated with a reduced risk of recurrent complicated peptic ulcers in ESRD patients. We recommend administering *H pylori* eradication within 120 days after a peptic ulcer diagnosis to *H pylori* infected ESRD patients with peptic ulcer disease.
